# Integrative physiological and transcriptome analysis of *Festuca rubra L.* in response to drought stress

**DOI:** 10.3389/fpls.2026.1771252

**Published:** 2026-04-13

**Authors:** Chun-Wei Sun, Zhao-Rui Xu, Li-Ping Zeng

**Affiliations:** School of Grassland Science, Beijing Forestry University, Beijing, China

**Keywords:** drought, *Festuca rubra* L. (F. rubra), peptide, transcription factors, transcriptome

## Abstract

Drought, as the primary abiotic stress constraining global agriculture and ecological stability, exerts a decisive impact on the survival and fitness of *Festuca rubra L.* (*F. rubra*) in arid and semi-arid habitats. Deciphering the molecular basis and regulatory networks underlying drought tolerance is essential for cultivation and genetic improvement of *F. rubra*. Here, we integrate the transcriptional analysis with functional analysis to elucidate the mechanisms response to drought. Pairwise comparison of drought-stressed, control, and rewatered samples revealed distinct transcriptional responses. A total of 1,296 DEGs were identified in the DS vs CK comparison, while 3,535 and 3,941 DEGs were detected in the RW vs DS and RW vs CK comparisons, respectively. Further analyses identified 222 transcription factors with putative roles in mediating drought resistance. Quantitative real-time PCR (qRT-PCR) confirmed the expression patterns of six selected DEGs, laying a foundation for subsequent functional investigations. Additionally, evolutionary analysis of the *CLE* families showed that *CLE25* is highly conserved in rice and Arabidopsis compared with *F. rubra*. The phenotype demonstrated that *CLE25*-mediated signaling pathway involving in regulating drought response in *F. rubra*. Our study was the first to study the regulatory mechanism of drought resistance of *F. rubra* with transcriptome methods and functional analysis identified the *CLE25*-mediated signaling pathway response to drought, which provides genetic resources for the breeding of drought-resistant varieties.

## Introduction

*Festuca rubra L.* (*F. rubra*), a high-quality forage and turfgrass grass, is widely cultivated across various ecological zones and is considered as a crucial grass species for maintaining terrestrial ecosystem stability. With global climate change, numerous studies have shown that drought has become more frequent and severe. Breeding drought-resilient grasses is recognized as a solution for mitigating the negative outcomes of drought and has become an important and urgent goal for global grass researchers (Valliyodan and Nguyen, 2006; Singh and Laxmi, 2015; Gong et al., 2020; Gampe et al., 2021).

Drought stress severely impacts plant growth and development. Plants initiate multi-dimensional response mechanisms under water deficit. Morphologically, shoot and leaf wilting becomes evident with water loss. Physiologically and metabolically, chlorophyll synthesis is inhibited while degradation accelerates, leading to a significant reduction in photosynthetic efficiency. Biochemically, excessive reactive oxygen species (ROS) accumulate in cells, damaging biomembranes, proteins, and nucleic acids. Key enzymatic protectants are superoxide dismutase (SOD), catalase (CAT), ascorbate peroxidase (APX), glutathione peroxidase (GPX), and peroxidase (POD). At the cellular level, plants regulate intracellular osmotic potential by accumulating organic compounds (e.g., proline, soluble sugars, betaine) and inorganic ions maintaining cell turgor and redox balance to mitigate drought-induced structural damage (Li et al., 2020). Drought stress ultimately causes growth retardation or arrest, as well as reduced yield and quality.

Moreover, plants cope with drought stress via various strategies, including the adjustment of metabolism and transcriptional regulation of stress-responsive genes. Abscisic acid (ABA) a key plant stress-signaling hormone, is accumulated. ABA also interacts with other hormones (e.g., ethylene, gibberellin, auxin, cytokinin, salicylic acid, and jasmonic acid) to regulate drought resistance (Chinnusamy et al., 2008; Waadt et al., 2022). And plants integrate drought signal transduction, photosystems, osmotic regulation, and antioxidant systems (Fang and Xiong, 2015), forming complex regulatory networks. Antioxidant enzyme genes, osmotic regulator synthase genes, and transcription factors (such as bZIP, AP2, EREBP, MYB and NAC) exert a crucial influence on this process, establishing multi-level regulatory networks (Bang et al., 2022; Jiang et al., 2012; Lim et al., 2022; Rushton et al., 2020; Xianjun et al., 2011; Xiong et al., 2025; Yang et al., 2005; Zhu, 2016).

Up to present, the *CLE* (*CLAVATA3*/Embryo Surrounding Region-related) family is the largest molecular family of plant peptides, which plays a key role in response to stress (Sawa et al., 2008; Shigeyuki et al., 2011; Murphy et al., 2012). In *Arabidopsis thaliana*, there are 32 *CLE* family genes encoding 27 unique peptides (Czyzewicz et al., 2015). *CLE* genes have been reported to involving in various biological processes, including plant growth, development and responses to stress (Fiers et al., 2005; Kondo et al., 2011; Kinoshita et al., 2007; Qian et al., 2018; Gupta and Sawa, 2019; Zhang et al., 2019; Fletcher, 2020; Zhang et al., 2022; Yang et al., 2025).

The development of high-throughput sequencing technologies has facilitated the mining of drought-responsive genes in various plants, greatly advancing research on stress resistance mechanisms. Based on this, transgenic and gene-editing technologies targeting drought-resistant genes have improved plant drought tolerance (Stark et al., 2019; Gupta et al., 2020). Transcriptomic studies on heat tolerance, salt-alkali resistance, and tillering traits in *Lolium arundinaceum* (tall fescue) have been reported (add references). However, no genomic or transcriptomic studies on *F. rubra* response to drought has been published to date. Therefore, this study aims to identify regulatory genes and excavate stress-resistant gene resources in *F. rubra* under drought stress using transcriptomic approaches.

## Materials and methods

### Plant materials, growth conditions and drought stress treatment

The germinated seeds of *F. rubra* (Mengshen, Beijing Zhengdao Seed Industry Company) were individually sown in separate pots containing a sterilized substrate mixture (vermiculite: potting soil, 1:3, v/v). Prior to sowing, the substrate was fully saturated to field capacity with deionized water. The pots were incubated in a controlled-environment growth conditions with16 h light/8 h dark photoperiod, 23°C/20°C day/night temperature regime, and a relative humidity of 60% ± 5%. Throughout the cultivation period, deionized water was supplied daily at a fixed volume to maintain the substrate water content consistently above 80% of field capacity. At 36 days after sowing, individual pots were weighed and then transferred to dry trays. Pots were weighed daily to calculate the relative substrate water content (RSWC). Drought stress treatment was officially initiated when RSWC decreased to 30%, designated as day 1 of drought stress. For rewatering treatment, plants were rehydrated for 1 day, with the substrate water content maintained at above 80% of field capacity.

For each treatment group, the aboveground tissues of one intact *Festuca rubra* plant were harvested as a single biological replicate. Sampling was performed at 12:00 noon, corresponding to 4 hours after the onset of illumination in the controlled-environment growth conditions. Plant samples were separately collected from three groups: the 53-day-old seedlings of drought-stressed group, the well-watered control group, and the 1-day rewatered group (with substrate water content maintained above 80% of field capacity; sampling at 12:00 noon, 4 hours after light initiation in the growth chamber). Three biological replicates were established for each group. Seedlings were collected from individual plants in the control and treatment groups seedlings of drought stress and rewatering. Samples were immediately frozen in liquid nitrogen and stored at -80 °C. Leaves from three individual plants were used as three biological replicates.

### High-throughput sequencing

Total RNA was extracted from *F. rubra* leaf tissues using Trizol reagent. The RNA was isolated to construct a non-strand-specific RNA-seq transcriptome sequencing library, which was sequenced on the BGI DNBSEQ-T7 platform by Annoroad Gene Technology. Each library generated over 10 Gb of valid data.

### Data filtering, assembly, and annotation pipeline

Low-quality reads and adapter sequences were first removed (Bolger et al., 2014). Filter the low-quality reads by Trimmomatic software with the length threshold of 100 nt and quality threshold of 20 (LEADING:20 TRAILING:20 MINLEN:100 SLIDINGWINDOW:3:20). Transcriptome assembly was performed using Trinity-v2.12.0, and the longest contig set from the assembly was selected. The TransDecoder. LongOrfs module was used to identify the longest open reading frames (ORFs) in transcripts. Domain prediction was conducted via the hmmscan module in HMMER 3.3.2 based on the Pfam (v35) database. Meanwhile, proteins were predicted using TransDecoder. Predict 5.7.1, which integrated domain annotations from HMMER and blastp (BLAST 2.15.0+) alignments against the UniProt database (Release 2024_05). All predicted genes were functionally annotated by aligning with the eggNOG (v5.0.2) database using emapper-2.1.12 (Cingolani et al., 2012; Bolger et al., 2014).

### Differential expression analysis and gene enrichment analysis

High-quality reads from each sample were aligned to CDS sequences using RSEM-1.2.31 to generate gene expression matrices. Differentially expressed genes (DEGs) were identified using DESeq2 embedded in TBtools-II (v2.136) for three comparisons: control/drought, control/rewatering, and drought/rewatering. The detailed procedures are as follows:

1. Filtered the genes with sum of mapped reads less than 10 reads. And genes between two groups with expression level |log2foldchange|≥1 and adjusted threshold of 0.01 were taken as DEGs.
2. |log2foldchange threshold|≥1 and adjusted p-value cutoff of 0.01
3. Multiple testing correction was performed using the Benjamini–Hochberg method, with a false discovery rate (FDR) threshold set at < 0.01.
4. The longest transcripts were used for CDS prediction and expression level analysis. Therefore, all the analysis were based on gene level.

Gene Ontology (GO) enrichment analysis was performed using TBtools following a standardized pipeline. Briefly, three core files were prepared: the go-basic.obo database (downloaded from the official GO repository), a genome-wide gene-GO term annotation file (multiple GO terms per gene separated by commas), and a query gene list (one gene ID per line). Stringent quality control was conducted to ensure consistent gene ID nomenclature across files, with empty lines and duplicates removed. Next, the GO Enrichment module was launched, and the database, background annotations, and query list were imported sequentially. Key parameters were set to high standards: Fisher’s exact test for statistical analysis, Benjamini-Hochberg (BH) correction for multiple testing, and significance thresholds of false discovery rate (FDR) < 0.05 and enrichment factor ≥ 1.5, with a minimum of 5 genes per enriched GO term to exclude small-set false positives. For bubble plot visualization, the x-axis represented enrichment score and the y-axis represented GO terms ranked by *P*-value; bubble size was proportional to the number of annotated genes per term, and bubble color indicated *P*-value magnitude (Chen et al., 2020; Mu et al., 2024).

### Synthesis of peptide and spraying assay

The *CLE25* peptide (RRVPNGPDPIHN) was synthesis in GenScript (0.44 mmol/g, 0.2 mmol scale). The Seedlings were grown under 16h light/8h dark. Following a 31-day growth period, drought stress was simulated by withholding irrigation for 19 days. Three biological replicates were set up, and plants were randomly assigned to two groups: the treatment group received foliar application of the peptide, while the control group was sprayed with an equivalent volume of deionized water, followed by gene expression analysis. Concentration of gradient pf *CLE25*(0,2.5,5.0,7.5, and 10 uM) were using for exogenous spraying. The concentration between 7.5 uM to 10.0 uM showing similar effectiveness. Therefore, we used water and the peptide solution with the concentration of 10 uM in the following experiment. We sprayed the solution every 2 days. Spraying of the peptide was conducted following light exposure in the morning, and plant samples were harvested at 1 h, 3 h, and 5 h after spraying to determine the corresponding gene expression profiles.

### Measurements of proline, malondialdehyde contents and antioxidant enzyme activities in *F. rubra* Leaves

Frozen leaves of *F. rubra* were homogenized in a chilled mortar and pestle with 50 mM potassium phosphate buffer (pH 7.8) containing 1 mM ethylene diamine tetraacetic acid (EDTA), 3 mM 2-mercaptoethanol, and 2% (w/v) polyvinylpolypyrrolidone (PVPP). The homogenate was centrifuged at 15,000 × g for 30 min at 4 °C. The supernatant was then used for subsequent assays.

Superoxide dismutase (SOD) activity was measured using the nitroblue tetrazolium (NBT) method (Dhindsa et al, 1981; Hodges and Forney, 2000). Absorbance was measured at a wavelength of 560 nm, with 50% inhibition as one unit. Blanks (no illumination) and controls (no enzyme) were processed in parallel under the same conditions.

Enzyme activities were determined with the method described previously (Li et al, 2017). CAT activity was monitored at 240 nm. One unit of CAT activity was defined as an absorbance change of 0.01 units per minute. Absorbance of the reaction solution at 470 nm was measured every 30 second, and one unit of POD activity was designated as an absorbance change of 0.01 units per minute.

For the determination of malondialdehyde (MDA) we used the method described previously at 532 and 600 nm absorbance (Zhang et al, 2011). The proline (Pro) content was calculated via the sulfosalicylic acid method at 520 nm with a standard curve (Bates et al., 1973).

### Reverse transcription quantitative PCR

Primers were designed using Primer Premier 5.0 based on gene sequences. To validate RNA-seq results, six transcription factors were selected from DEGs, and primers were designed according to their CDS sequences. *Festuca rubra* actin was used as the internal reference gene (**Supplementary Table 1**).

Total RNA was isolated from the leaves of *F. rubra*. Using 500 ng RNA as the template, cDNA was synthesized via reverse transcription with the Takara RT-PCR Kit (Takara Biomedical Technology, Dalian, China). Quantitative real-time PCR reactions were set up using the TB Green™ Premix Ex Taq™ II kit (Takara, Dalian, China) on a CFX96 C1000 qPCR instrument (Bio-Rad, USA). The 20-μL qPCR reaction system included: 10 μL reaction mixture, 1 μL of each forward/reverse primer, 2 μL cDNA, and 6 μL sterile water. Amplification was performed on a CFX96 C1000 qPCR instrument (Bio-Rad, USA) with the following program: 95 °C for 30 s (initial denaturation), followed by 40 cycles of 95 °C for 5 s and 60 °C for 30 s. The Relative gene expression was calculated using the 2-△△Ct method, with three technical replicates for each biological sample.

## Results and analysis

### Drought stress induced morphological and physiological changes in *F. rubra*

To investigate the adaptive strategies of *F. rubra* in response to drought stress, we characterized its phenotypic and physiological alterations under drought stress. Drought stress induced adaptive remodeling of morpho-structural traits in *F. rubra*, aiming to optimize water retention efficiency and light energy utilization under water-deficit conditions. Specifically, the plants exhibited leaf wilting and drooping, coupled with a reduction in leaf area, which lowered the rate of water loss by shrinking the effective transpiration surface area.

**Figure 1:**
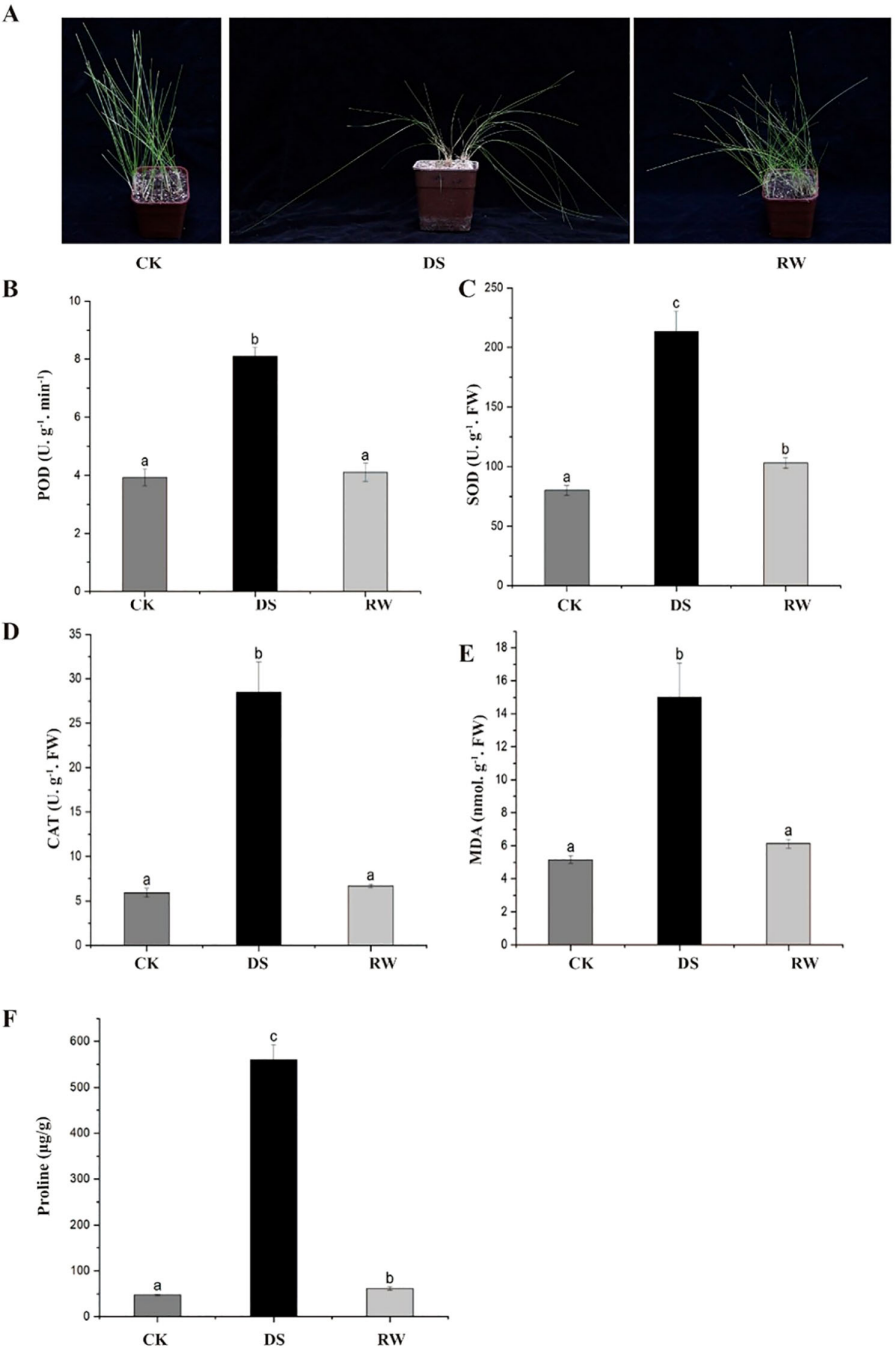
Phenotypic and physiological responses of plants under control, drought stress and rewatering conditions. **(A)** The phenotype of *F. rubra* change in well-waterd control (CK), drought treatment (DS) and rewatered (RW) groups. **(B-D)** Activity of peroxidase (POD), superoxide dismutase (SOD) and catalase (CAT). **(E)** Content of malondialdehyde (MDA). **(F)** Content of proline. The values are calculated with three independent biological replicates. Error bars represent the standard deviation. ANOVA Tukey’s multiple comparisons test was applied, and letters represent the statistical differences among groups (P < 0.001).

Upon rewatering, *F. rubra* exhibited high-efficiency morpho-structural plasticity, with wilted and drooping plants rapidly recovering to an upright growth state; notably, the recovery rate was negatively correlated with the duration of drought stress—the longer the drought exposure, the slower the recovery process (**Figure 1A**).

Under drought stress, excessive ROS accumulation can induce oxidative damage to cellular components, particularly lipids, leading to membrane peroxidation. Our results showed that compared with the non-stressed control and rewatering plants, the Malondialdehyde (MDA) content in drought stressed plants were significantly elevated (**Figure 1E**). Concomitantly, the activity of the key tested antioxidant enzymes was significantly upregulated under drought stress, including SOD, CAT and POD (**Figures 1B–D**). Besides, the contents of proline in *F. rubra* were significantly increased under drought stress compared with control and rewatering plants, serving as osmoprotectants to preserve cell turgor and alleviate dehydration-induced damage (**Figure 1F**). Taken together, the phenotypic and physiological responses of *F. rubra* to drought stress define a coordinated defense system, providing a critical physiological basis for elucidate the transcriptional regulation mechanisms.

### Transcriptional dynamics of *F. rubra* Leaves under drought stress and subsequent rewatering

A total of 9 transcriptome libraries were constructed and subjected to high-throughput sequencing, corresponding to three biological replicates of each group (CK, DS and RW) Sequencing yielded 903,704,268 raw reads in total. After filtering adapter-containing and low-quality reads (N >5%), 867,192,644 clean reads were retained, with a valid rate of 95.96%. *De novo* assembly of clean data generated 108,514 unigenes with an N50 length of 1,401 bp. The total sequence length was 128,500,514 bp and the GC content was about 50% (**Supplementary Tables 2**, **S3**).

**Figure 2:**
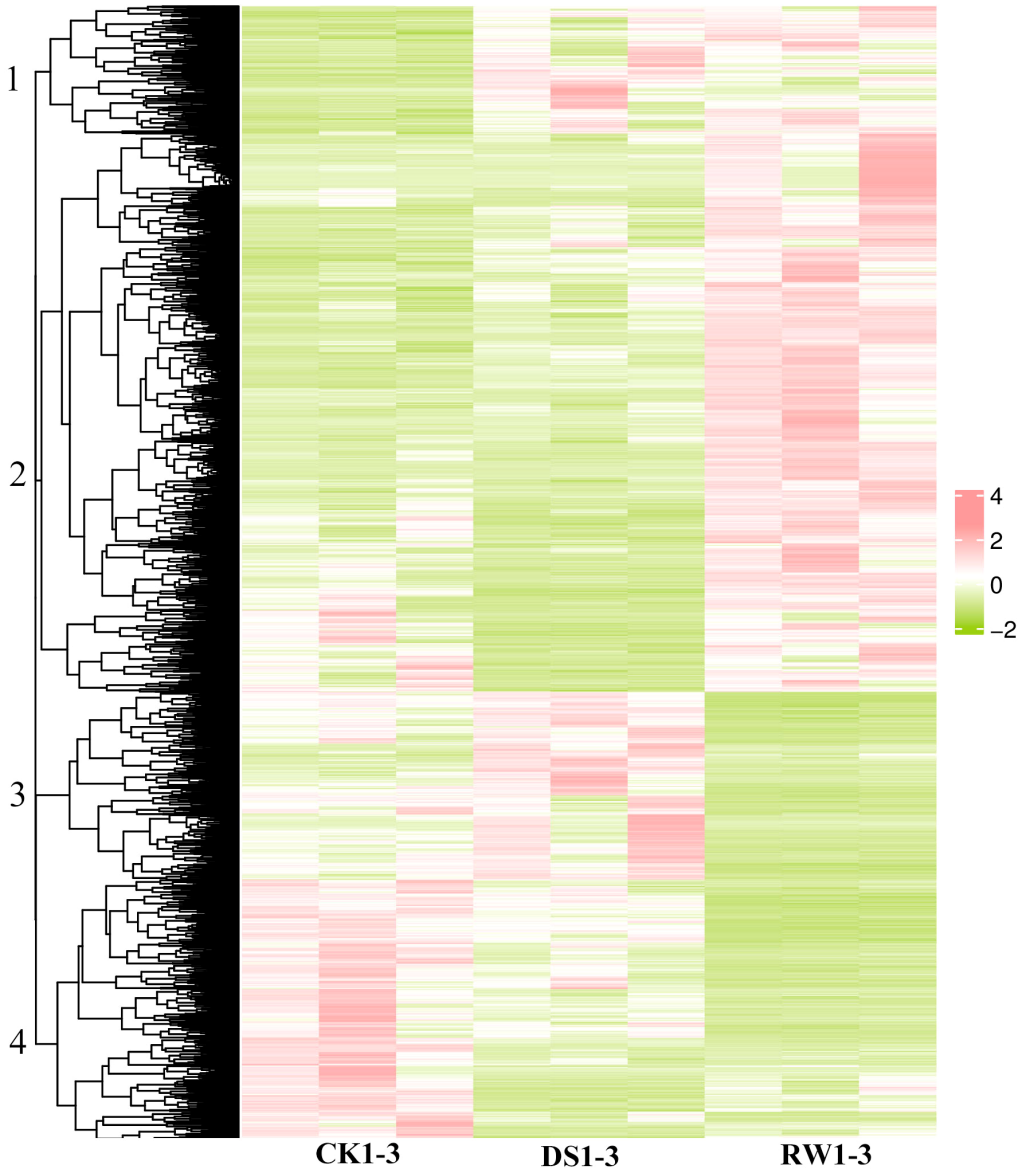
Heat map of the differentially expressed genes. The abbreviations CK, DS and RW correspond to the control, drought stress and rewatered groups, respectively. Z-scores are used to compare expression levels between samples.Each group includes three independent biological replicates for RNA-seq expression profiling.

Pairwise comparisons were performed among three experimental groups to identify differentially expressed genes (DEGs), with the transcriptional dynamics distinctly reflected by the patterns of gene upregulation and downregulation across drought-stressed versus control (DS vs CK), rewatering versus drought-stressed (RW vs DS), and rewatering versus control (RW vs CK) groups (**Supplementary Table 4**). Specifically, in the DS vs CK comparison, a total of 1,296 DEGs were identified, among which 788 were significantly downregulated and 508 were upregulated; the RW vs DS comparison yielded 3,535 DEGs, including 1,200 downregulated and 2,335 upregulated candidates; and 3,941 DEGs were detected in the RW vs CK comparison, with 1,427 being significantly downregulated and 2,514 upregulated (**Figure 2**; **Supplementary Table 4**).

**Figure 3:**
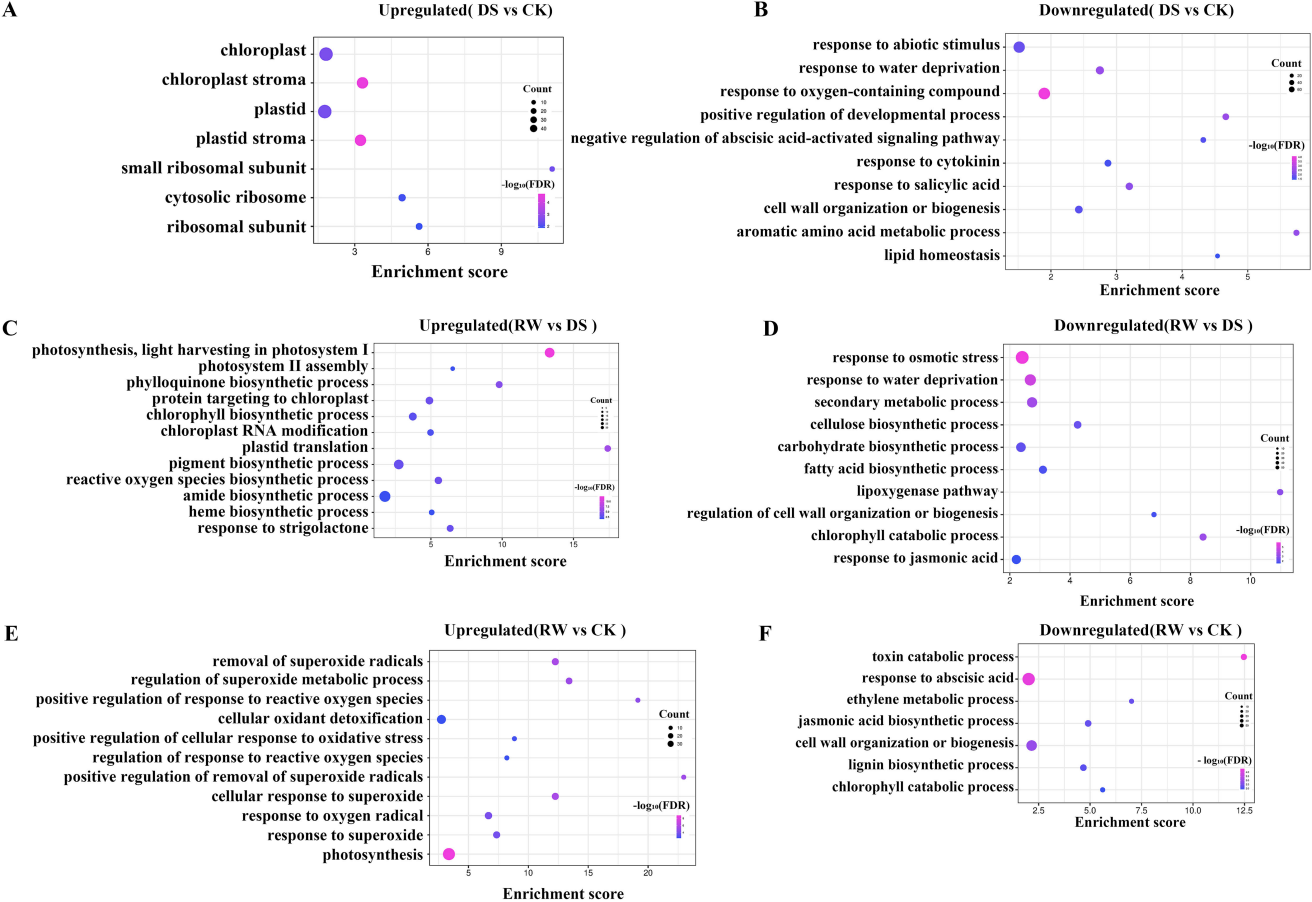
Transcriptomic characteristics of **(F)** rubra in response to drought stress and rehydration. **(A)** GO analysis of 508 upregulated DEGs between the well-watered control (CK) and drought treatment (DS). **(B)** GO analysis of 788 downregulated DEGs between the well-watered control (CK) and drought treatment (DS). **(C)** GO analysis of 2335 upregulated DEGs between the drought treatment (DS)and rewatered (RW) groups. **(D)** GO analysis of 1200 downregulated DEGs between the drought treatment (DS) and rewatered (RW) groups. (**(E)** GO analysis of 2514 upregulated DEGs between well-watered control (CK) and rewatered (RW) groups. **(F)** GO analysis of 1427 downregulated DEGs between well-watered control (CK) and rewatered (RW) groups. Horizontal axis: enrichment score; vertical axis: enriched pathway names; color scale: P-value thresholds; dot size: gene count per pathway.

In DS vs CK, upregulated genes mediate chloroplast physiological processes under drought stress (**Figure 3A**; **Supplementary Table 5**). Conversely, downregulated genes were significantly enriched in pathways associated with water deprivation, cell wall biogenesis, hormone signaling (cytokinin/salicylic acid responses and negative regulation of abscisic acid-activated signaling), and homeostasis of lipids and aromatic amino acids (**Figure 3B**; **Supplementary Table 5**).

In RW vs DS, upregulated genes were mainly associated with photosynthesis, including light harvesting in photosystem I and photosystem II assembly, alongside phylloquinone, chlorophyll, pigment, amide, and heme biosynthetic processes, while also directing protein targeting to chloroplasts, chloroplast RNA modification, plastid translation, reactive oxygen species biosynthesis, coupled with response to strigolactone (**Figure 3C**; **Supplementary Table 5**). Downregulated genes were significantly enriched in response to osmotic stress, response to water deprivation, cellulose and carbohydrate biosynthetic processes, fatty acid biosynthetic processes, lipoxygenase pathways, regulation of cell wall organization or biogenesis, and response to jasmonic acid (**Figure 3D**; **Supplementary Table 5**).

In RW vs CK, upregulated genes were predominantly enriched in photosynthesis, alongside regulation of superoxide metabolic process, positive regulation of response to reactive oxygen species and cellular oxidant detoxification (**Figure 3E**; **Supplementary Table 5**). The downregulated genes were significantly enriched in processes related to toxin catabolic processes, hormone signaling, including abscisic acid responses, ethylene, and jasmonic acid metabolic pathways, representing a regulatory strategy to facilitate growth normalization (**Figure 3F**; **Supplementary Table 5**).

Collectively, comparative transcriptomic analyses revealed that drought triggers adaptive transcriptional changes centered on chloroplast physiology and stress-related hormone signaling (ABA, cytokinin, and salicylic acid). Upon rewatering, a global transcriptional shift occurs toward photosynthetic recovery, reactive oxygen species detoxification, hormone signaling (ABA, ethylene, jasmonic acid, and strigolactone) and the downregulation of stress-responsive pathways.

### Transcriptional profiling dynamics of key transcription factors in *F. rubra* in response to drought stress and rewatering recovery

**Figure 4:**
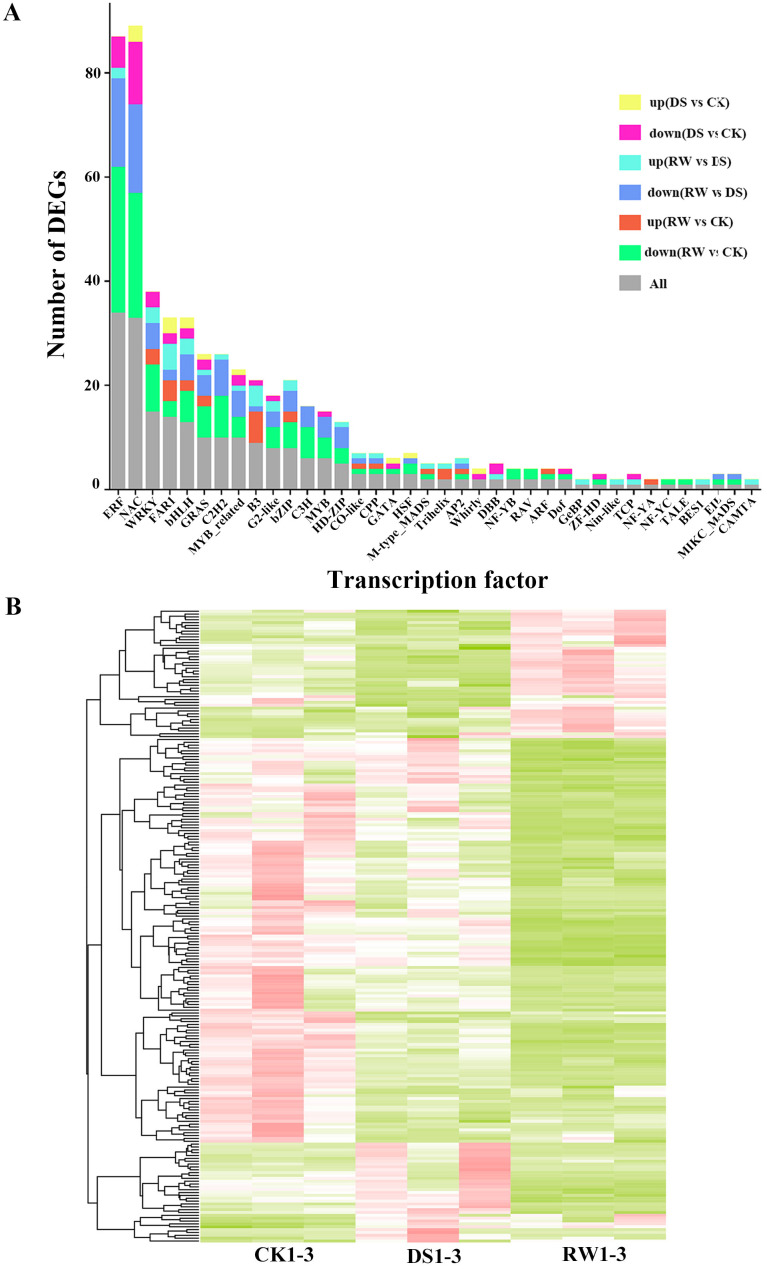
Transcriptomic profiles of transcription factors (TFs) in *F. rubra* in response to drought stress and rehydration. **(A)** The gene family distribution of the differentially expressed transcription factor families among CK, DS and RW treatments. **(B)** Heatmap of transcription factor-encoding genes across three experimental groups: CK (well-watered control), DS (drought-stressed), and RW (post-drought rewatered). Z-scores are used to compare expression levels between samples.

Transcription factors (TFs) accounted for merely 1.90% of the total transcribed genes, whereas their proportion surged to 3.48% among DEGs, indicating that drought stress preferentially perturbs TF expression. The total number of transcription factors across the three comparisons (DS vs CK, RW vs DS, and RW vs CK) was 222 among DEGs genes of *F. rubra*. The top 3 most abundant transcription factor families were ERF (34 members), NAC (33 members), and WRKY (15 members), accounting for 15.32%, 14.86%, and 6.76% of the total differentially expressed transcription factors, respectively. Other prominent families included FAR1 (14), bHLH (13), GRAS (10), C2H2 (10), and MYB_related (10). The remaining TF families, such as B3, G2-like, bZIP, C3H, MYB, and HD-ZIP, contained fewer members (≤9), while several families (e.g., GeBP, ZF-HD, Nin-like, TCP) were represented by only a single member each. For the NAC and WRKY TF families, under drought stress,the number of genes with reduced transcript levels was markedly higher than that in the control group. Following rewatering, the transcriptional profile of NAC and WRKY TFs maintained a predominantly downregulated expression pattern (**Figures 4A, B**; **Supplementary Table 6**).

In DS vs CK, 13 and 39 transcription factors were up‑ and down‑regulated, respectively. In RW vs DS, these numbers were 36 and 88; in RW vs CK, 27 and 128. The number of downregulated TFs was higher than that of upregulated TFs in most comparison groups. Notably, the greatest number of differentially expressed TFs was observed in the RW vs CK comparison. The downregulated sets RW vs CK, contained the most abundant transcription factors, whereas those up regulated sets in CK vs DS were fewest. Among the three sets of down-regulated differentially expressed genes, 8 were shared, including 2 NAC, 3 ERF, 2 C2H2 and 1 WRKY, which represent the core transcription factor families responsive to drought stress and rewatering. Among them, the ERF transcription factor (TRINITY_DN47016_c0_g1_i1.p1, AT4G25490.1) belongs to the ERF family and *Arabidopsis* homolog has been reported to respond to ABA, while the WRKY (TRINITY_DN10562_c0_g1_i14.p1, AT4G11070.2) and C2H2 (TRINITY_DN10663_c1_g1_i18.p2, AT1G27730.1) TFs of *Arabidopsis* homolog are involved in stress tolerance (**Figure 4A**; **Supplementary Table 6**).

**Figure 5:**
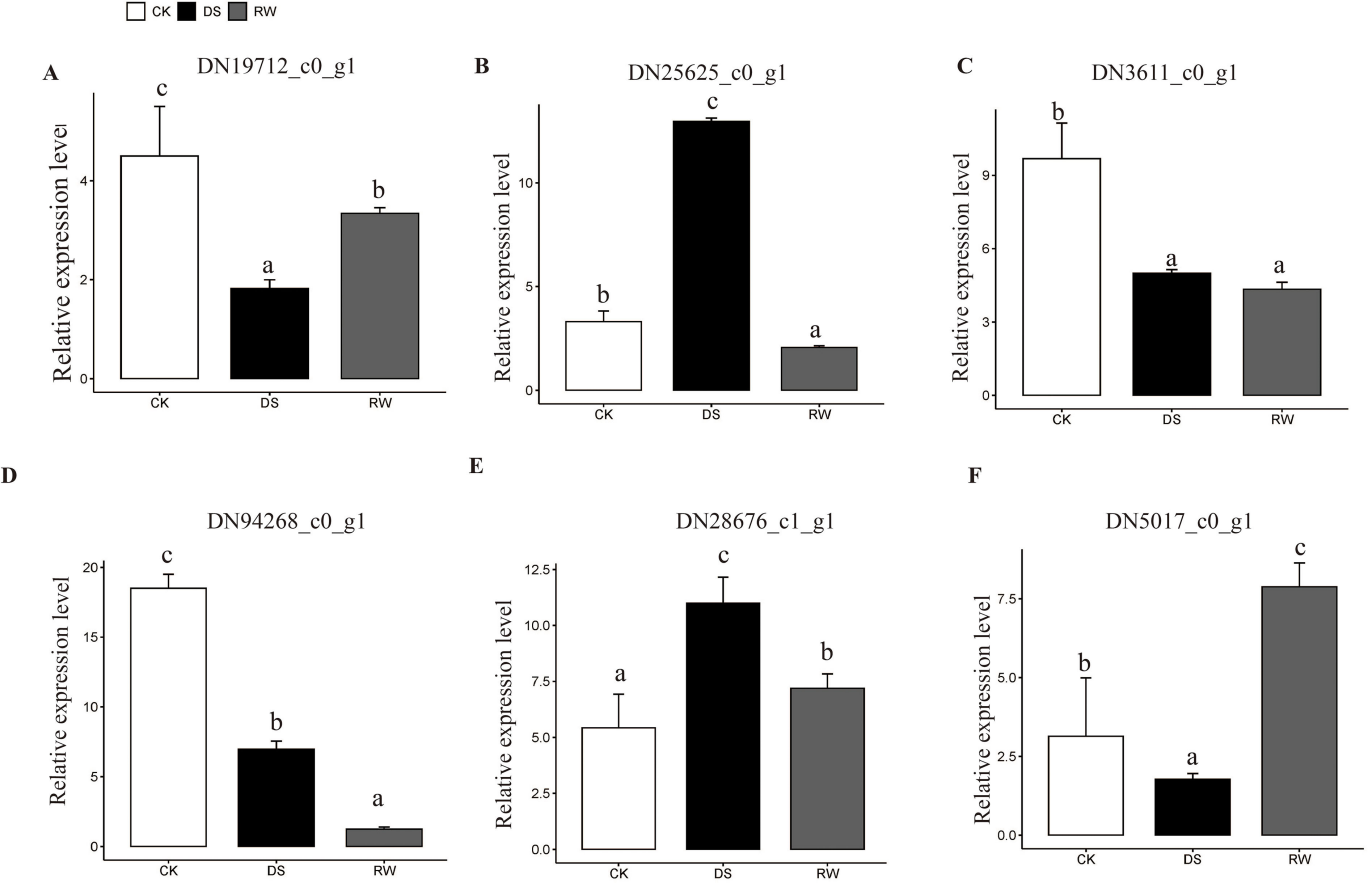
Expression verification of differentially expressed transcription factor using qRT-PCR. **(A–F)** depict the transcriptional dynamics of six key transcription factors in Festuca rubra under drought stress and rewatering: **(A)** DN19712, an ERF transcription factor; **(B)** DN25625, a member of the NAC transcription factor family; **(C)** DN3611, belonging to the WRKY transcription factor family; **(D)** DN94268, a C2H2-type transcription factor; **(E)** DN28676, a FAR1-family transcription factor; **(F)** DN5017, a G2-like transcription factor. The abbreviations CK (white), DS (black) and RW (gray)correspond to the control, drought resistant and rewatered groups, respectively. The relative expression level of each gene was calculated using actin as the reference gene, with data obtained from three independent biological replicates. Error bars represent the standard deviation. ANOVA Tukey’s multiple comparisons test was applied, and letters represent the statistical differences among groups (P < 0.001).

The relative expression of *DN25625* (NAC TF; *Arabidopsis* homolog AT3G04070) was significantly higher than the control under drought stress but lower after rewatering. *DN28676* (FAR-1 TF; *Arabidopsis* homolog AT4G38180.1) exhibited significantly higher expression than the control under drought stress, with slightly higher expression after rewatering. *DN3611*(WRKY TF; *Arabidopsis* homolog was AT3G58710.1) and *DN94268* (C2-H2 TF; *Arabidopsis* homolog AT3G49930.1) showed lower expression than the control under both drought stress and rewatering. *DN19712* (ERF TF; *Arabidopsis* homolog AT3G16770.1) and *DN5017* (G2-like TF, *Arabidopsis* homolog AT3G13040.1) had lower expression under drought stress but higher expression after rewatering (**Figure 5**; **Supplementary Table 6**). These results confirm the differential drought and rewatering-responsive expression patterns of the candidate genes and validate the reliability of the RNA-seq dataset for further molecular mechanism dissection.

### The mature *CLE25* peptide mediates drought tolerance via the ABA biosynthsis pathway in *Festuca rubra*

It has been reported that *CLAVATA3* (*CLV3*)/endosperm surrounding region (*CLE*) peptides are involved in plant growth and development (Cock and McCormick, 2001), as well as in the response to abiotic stresses (Takahashi et al., 2018; Datta et al., 2024). However, due to the lack of genomic and transcriptomic resources in *F. rubra*, the functional roles of *CLE* genes in this species remain uncharacterized.

**Figure 6:**
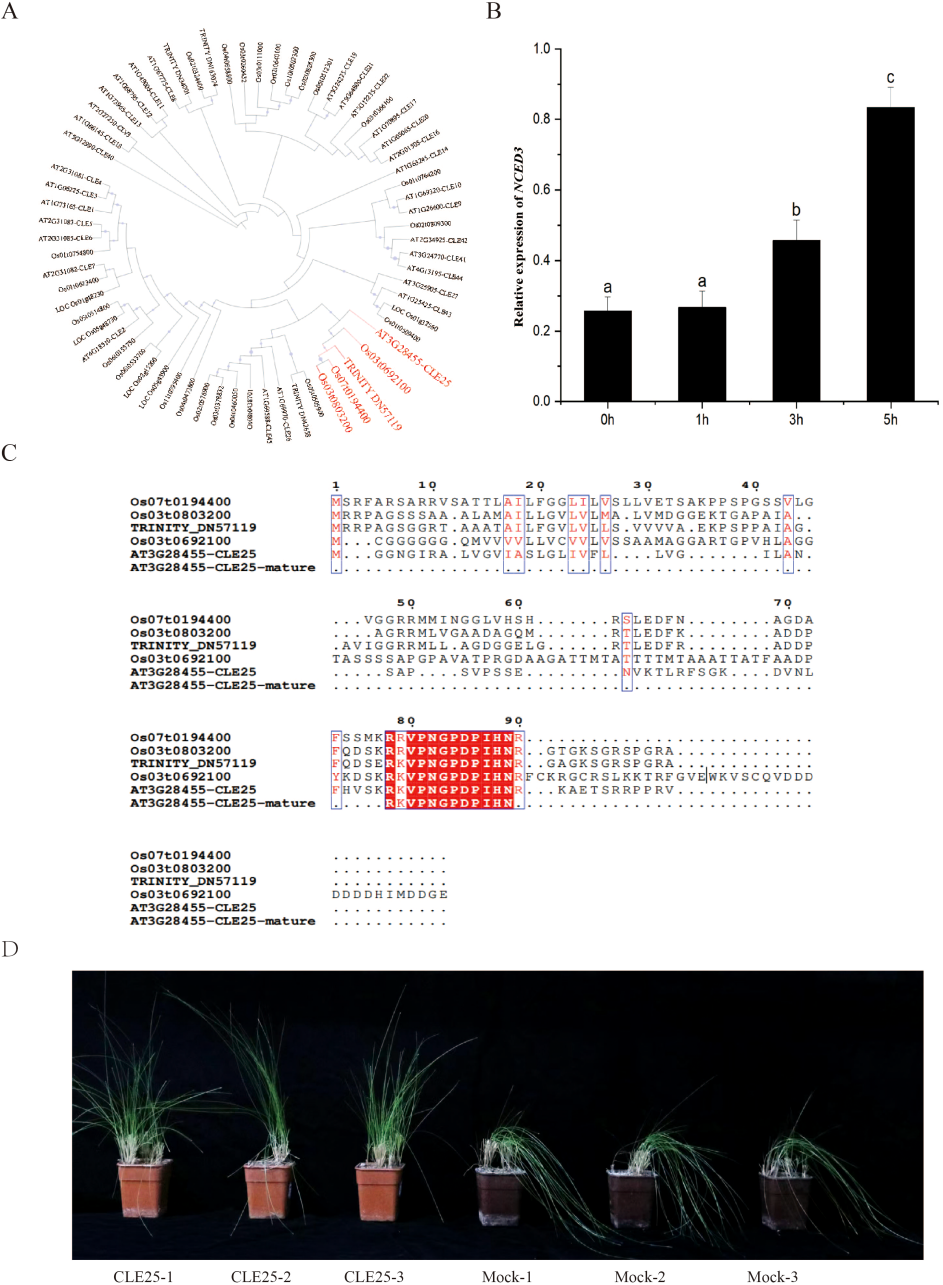
*CLE25* enhances *F. rubra* drought resistance. **(A, C)** phylogenetic tree of *CLE* family peptides were constructed using the maximum likelihood method. The evolutionary conservation of *F. rubra CLE25* and its homologous *CLE* peptides from *Arabidopsis thaliana* and *Oryza sativa* was analyzed. **(B)** The expression of ABA pathway genes *NCED3* in *F. rubra* plants induced by 10 μM *CLE25* for 1h, 3h and 5h. **(D)** Phenotypic comparison of *F. rubra* seedlings sprayed with Mock (H_2_;O) vs. 10 µM *CLE25p* under drought stress. Seedlings were cultivated under a 16 h light/8 h dark photoperiod. After 31 D(days) of growth, drought stress was induced by 19 D (days) irrigation withholding. Drought-treated seedlings were then sprayed with H_2_;O (mock) or 10 µM *CLE25p* at 2 D (days) intervals, with three biological replicates per treatment. Error bars represent the standard deviation. ANOVA Tukey’s multiple comparisons test was applied, and letters represent the statistical differences among groups (P < 0.001).

We focused on the *CLE25* gene, which has been reported as drought-response-associated *CLE* genes in *Arabidopsis thaliana* (Fletcher, 2020). Firstly, we identified homologous *CLE25* gene from the *F. rubra* transcriptome assembly generated in this study, followed by a comparative evolutionary analysis among *Oryza sativa*, *Arabidopsis thaliana* and *F. rubra*. Notably, the *F. rubra* precursor *CLE25* protein clustered with its *Arabidopsis thaliana* ortholog, while the mature *CLE25* peptide of this species has an identical amino acid sequence with that of *Arabidopsis thaliana* (**Figures 6A, C**). Given this conserved feature, we chemically synthesized the mature *CLE25* peptide and assayed its performances in mediating drought resistance.

After exogenous spraying of the mature *CLE25* peptide on wild-type plants, we found that this treatment significantly enhanced drought resistance compared with the control group. Concurrently, the expression level of *NCED3*(TRINITY_DN51594_c0_g1), a well-established marker gene for abscisic acid (ABA) biosynthesis, was also significantly upregulated (**Figures 6B, D**). Taken together, these results suggested that *CLE25* plays a positive role in mediating resistance to drought stress via the ABA biosynthesis pathway. Moreover, exogenous application of the mature *CLE25* peptide to alleviate drought stress holds substantial potential for practical application in *F. rubra*.

## Discussion

In recent years, with global climate change, drought is a significant factor affecting the environment and agricultural productivity (Riechmann et al., 2000; Singh et al., 2002; Yamasaki et al., 2008). Drought stress impairs the growth and productivity of *F. rubra*, it is therefore important to understand the *F. rubra* drought tolerance regulatory networks for advancing adaptive research and stress-resilient germplasm development. In this study, *F. rubra* were subjected to drought stress, during which key physiological and molecular responses were identified, including antioxidant defense enzyme activities, and genome-wide transcriptome profiles.

Drought–rewatering biological process analysis verifies that drought stress impairs photosynthetic function in *F. rubra*, a response conserved across plant species and validated by transcriptome data demonstrating differential gene upregulation in RW vs. DS and RW vs. CK comparisons. Simultaneously, drought elicits enhanced biosynthesis of stress-protective metabolites (e.g., proline), triggers antioxidant system activation to sustain redox homeostasis and upregulates peroxidase enzyme activities (e.g., POD and CAT); these synergistic responses scavenge excess ROS, mitigate membrane lipid peroxidation and maintain cellular structural integrity (Gupta et al., 2020), which matches functional annotation results from RW vs CK transcriptome analysis, with upregulated genes predominantly involved in superoxide radical elimination, reactive oxygen species response and oxidant detoxification pathways.

Pairwise transcriptomic comparisons of RW, DS and CK groups, coupled with differential gene enrichment analysis, reveal that multiple phytohormone signaling pathways (i.e., jasmonate, ethylene, cytokinin) are dynamically responsive to drought stress in *F. rubra*, with the ABA pathway acting as the dominant central regulatory hub (Schroeder et al., 2001; Jiang and Asami, 2018). As previously reported (Jossier et al., 2009), water-limited conditions elevate endogenous ABA levels in plants, which not only induces systemic protective changes against drought stress but also triggers activation of the canonical ABA–PYL–PP2C–SnRK2 signaling cascade via ABA accumulation. Upregulation of ABA biosynthesis genes, which is tightly correlated with the dynamics of endogenous ABA accumulation, enhanced drought tolerance in rice, maize and other plants (Kuromori et al., 2018), consistent with our findings in *F. rubra*.

The plant drought-responsive TFs modulate drought-related gene expression via specific binding to their cognate target DNA sites within gene promoters, thereby activating or inhibiting core transcriptional machinery to facilitate plant adaptation to drought stress (Jin et al., 2017). We identified 222 drought-responsive TFs belonging to 38 distinct families in *F. rubra* under drought stress. The transcription factors investigated herein represent evolutionarily conserved categories, whose core functions are relatively conserved across plant species. For WRKY TF families (*WRKY18*, *WRKY40*, *WRKY46* and *WRKY70*) have validated the critical roles in regulating drought stress resistance (Chen et al., 2010; Shang et al., 2010; Chen et al., 2017). Our current study extends these findings by characterizing their responsive patterns during plant exposure to drought stress and subsequent rehydration recovery in *F. rubra*. Among the identified NAC TFs, including *NAC1*, *NAC3*, *NAC6*, *NAC36* and *NAC87*, all were responsive to drought stress and subsequent rewatering; previous studies have demonstrated that *OsNAC6* enhances drought resistance in rice by promoting lateral root formation (Lee et al., 2017), *IbNAC087* modulates jasmonic acid (JA)-dependent drought tolerance in sweet potato (Li et al., 2024b), and NAC1 and *NAC3* improve drought resistance via activating reactive oxygen species (ROS)-scavenging enzyme-encoding genes or repressing ROS production-related genes (Fang and Xiong, 2015; Chen et al., 2023). MYB transcription factors (TFs) in *F. rubra* mediate stress responses, consistent with the functions of *MYB86* in wheat (*Triticum aestivum* L.) and *MsMYBH* in alfalfa (*Medicago sativa*) (Song et al., 2020; Shi et al., 2024). While the specific functional mechanisms of these TFs (bHLH, FAR1, ERF, bZIP, CO-like, G2-like, among others) in *F. rubra*—particularly in responses to drought stress and subsequent rewatering—remain to be further elucidated (Jin et al., 2017; Wei et al., 2022; Li et al., 2024a; Dong et al., 2020). However, due to the lack of genomic and transcriptomic data for *F. rubra* and the absence of reported successful construction of its genetic transformation system so far, the analysis of *F. rubra* transcription factors is lacking. In the future, it is necessary to improve data resources and genetic transformation technology systems to lay a foundation for in-depth functional analysis and mechanism exploration (Shelake et al., 2022; Hwarari et al., 2024).

In addition, *CLE*, the largest small peptide family in plants, plays an important role in regulating growth and development as well as stress adaptability (Whitford et al., 2008; Shinohara and Matsubayashi, 2010; Yamaguchi et al., 2016; Goad et al., 2017). *CLE25* peptides enhance plant stress tolerance in *F. rubra*. In the future, it is necessary to combine multi-omics technologies, gene editing, and protein-protein interaction analysis to systematically decipher the downstream regulatory networks and species-specific functional mechanisms of *CLE* peptides, providing novel molecular targets and theoretical support for the genetic improvement of *F. rubra* stress resistance.

Taken together, we used transcriptome sequencing technology to firstly analyze the types, quantities, and changes in expression levels of differentially expressed transcription factors in *F. rubra* under drought stress and rehydration conditions. In addition, CLE family’s analysis showed that *CLE25*-mediated signaling pathway involving in regulating drought response. Our results provide the genetic resources of functional genes regulating drought tolerance, thereby laying a molecular foundation for the rational design of drought-resistant *F. rubra* varieties and the extension of cultivation into water-limited regions, delivering profound implications for both the mechanistic elucidation and the promotion of agricultural ecological sustainability.

## Data Availability

The datasets presented in this study can be found in online repositories. The names of the repository/repositories and accession number(s) can be found in the article/**Supplementary Material**.

## Supplementary material

Supplementary Table 1 Genes and primer sequence for reverse transcription-polymerase chain reaction (RT-PCR).

Supplementary Table 2 The summary of RNA-Seq sequencing data.

Supplementary Table 3 Comprehensive statistics of expression levels for all detected genes.

Supplementary Table 4 Pairwise Comparison for DEG Screening (CK, DS and RW).

Supplementary Table 5 GO analysis and list of DEGs among the well-watered control (CK), drought treatment (DS) and rewatered (RW) groups.

Supplementary Table 6 Transcriptomic profiles of transcription factors (TFs) in *F. rubra* in response to drought stress and rehydration.
